# *The GOOD life*: Study protocol for a social norms intervention to reduce alcohol and other drug use among Danish adolescents

**DOI:** 10.1186/s12889-016-3333-1

**Published:** 2016-08-03

**Authors:** Christiane Stock, Lotte Vallentin-Holbech, Birthe Marie Rasmussen

**Affiliations:** Unit for Health Promotion Research, Department of Public Health, University of Southern Denmark, Niels Bohrs Vej 9-10, 6700 Esbjerg, Denmark

**Keywords:** Adolescents, Alcohol and other drug use, Social norms intervention, School, Denmark

## Abstract

**Background:**

It is currently unknown if school-based social norms interventions are effective in preventing harmful alcohol consumption and other drug use among adolescents in Denmark. This paper describes the social norms-based programme *The GOOD life* and the design of a cluster-randomized controlled trial to test its effectiveness.

**Methods/Design:**

The intervention *The GOOD life* is composed of three social norms components representing three different communication channels, namely face-to-face communication (normative feedback session), print communication (posters) and interactive media (web application). The intervention period of 8 weeks is preceded and followed by data collection, with the follow-up taking place 3 months after baseline. Public schools in the Region of Southern Denmark with grades 8 and 9 are invited to participate in the study and participating schools are randomly allocated to either intervention or control schools. The aim is to recruit a total of 39 schools and a sample of 1.400 pupils for the trial. An online questionnaire is conducted to examine the use of alcohol, tobacco and marijuana as well as the perceived frequency of use among peers of their own grade, which is measured before and after the intervention. Baseline data is used to develop social norms messages which are included in the three intervention components. Primary outcomes are binge drinking (more than 5 units at one occasion) and perceived frequency of binge drinking among peers, while smoking, marijuana use and alcohol-related harm will be assessed as secondary outcomes.

**Discussion:**

*The GOOD life* study will provide necessary insights on descriptive and injunctive norms regarding alcohol and other drug use among Danish adolescents. In addition, it will provide new knowledge and insight on the feasibility, implementation context and effectiveness of a newly developed social norms intervention in the Danish school context.

**Trial registration:**

Date of registration: 17 February 2016 (retrospectively registered) at Current Controlled Trials with study ID ISRCTN27491960

## Background

The use of alcohol among adolescents in Denmark is one of the highest in Europe, which applies both to quantities consumed at each drinking session as well as to the frequency of sessions [1–3]. Approximately 37 % of Danish pupils aged 15–16 years reported drunkenness in the last 30 days, while the European average for this age group is 17 % [2]. Similarly, the percentage of binge drinkers in this age group, i.e. those who have consumed five or more drinks at one drinking session in the last 30 days, is high with 60 % for boys and 53 % for girls, respectively [2]. Regarding other drug use, 6 % of Danish adolescents reported cannabis use within the last 30 days and an average of 5 % reported lifetime use of illicit drugs other than cannabis. Hence Danish adolescents reported a similar prevalence compared to adolescents in other European countries, where the average in 36 countries was 7 % and 6 %, respectively. Further experience with various drug use other than alcohol is more frequently reported among boys [2, 4].

Excessive alcohol use has serious short-term consequences for a number of young Danes. Among other harmful consequences, one third of boys and girls performed poorly at school due to drinking. Furthermore, a proportion of 17 % of boys have gotten into fights with peers as a result of alcohol use [5]. Adolescents who use alcohol, tobacco or other drugs achieve lower grades, have more negative attitudes towards school and exhibit increased absenteeism [6, 7]. A large longitudinal study in the US concluded that by the end of high school, alcohol consumption predicted declining socio-economic functioning with negative implications for adolescents’ academic grades [8]. Additionally, an increased likelihood of harmful drinking in adolescence has been described as contributing to long-term consequences with regard to harmful drinking as an adult [9]. It has also been observed that an early debut of alcohol drinking increases the risk of alcohol dependency and alcohol related diseases later in life [10, 11]. Thus, it can be argued that decreased substance use would reduce the risk of substance related events and other harmful behaviour, and potentially improve health and life prospects among young people.

Individuals, particularly young people, are strongly influenced by their perceptions of the behaviour and attitudes of those around them, which has been suggested by social comparison theory [12, 13]. However, it has also been demonstrated that individuals overestimate the risk behaviours of their peers (descriptive norms), and also how accepting their peers are of such behaviours (injunctive norms). The origins of this finding derive from evidence exploring alcohol use among students in the American college system [14–19]. Yet misperceptions of peer alcohol and other related substance use have also been documented in school and college students across a number of European countries [20–23].

This tendency towards misperception has serious consequences as individuals are likely to be driven to match what they perceive to be the norm amongst their peers, as suggested by psychological theories of behaviour such as the Theory of Planned Behaviour [24]. It is also known that individuals often lack awareness of how easily influenced they really are by the norms of a particular group [25]. Other social psychological research investigating factors such as attribution bias helps to explain how people tend to perceive those around them as behaving in a more risky way than themselves [26]. Such misperceptions may also be reinforced by media rhetoric which frequently perpetuates negative stereotypes. For instance, media portrayals in Denmark often describe adolescents and young people as regular and frequent heavy drinkers, despite the fact that in reality the majority of Danish adolescents do not binge drink on a regular basis [27].

The findings that reveal the presence of such misperceptions has become the basis for an approach towards behaviour change and prevention known as the social norms approach. This approach works on the premise that if misperceptions about a group norm are challenged, the social pressure on the individual to behave in the respective risky manner will decrease and result in the promotion of more positive, healthy behaviour [28]. Similarly, the social norms approach can be used to challenge misperceptions that people may hold regarding the attitudes of their peers, such as an individual perceiving their peers to support cannabis use to a higher extent than they actually do [29]. The social norms approach differs from other prevention approaches because it does not rely on negative or fear based imagery and it does not contain any moralistic messages about how the target population should behave or what their attitudes should be. Instead, it aims to empower individuals through promoting informed decision making by encouraging them to question the negative misperceptions they hold about their peers [22]. In conclusion, the social norms approach differs fundamentally from many other forms of prevention and behaviour change interventions by focusing on the positive behaviour of the majority, rather than on the negative behaviour of the minority.

It is increasingly evident from various studies that mass media campaigns with a moralistic or fear arousing focus have modest effects on young people and a few studies even report their negative effect. These counter-productive interventions often include a single component of many, and focus on the negative health consequences of a risk-behaviour [30–32]. When using social norms marketing to influence the behaviour of young individuals, it is essential to ensure sufficient repetition of the message through different channels such as posters, websites/web applications, flyers, e-mail messages and newspaper editorials. Furthermore, the setting in which the social norms marketing campaign is received should meet the needs, expectations and cultural requirements of the target group [30, 33]. In their umbrella review, Jepson et al. [32] found that the most effective interventions included school-based activities promoting positive behaviours and social norm marketing has been shown to be an effective method in decreasing alcohol consumption among adolescents [14, 15].

A European study with 170 intervention and control schools investigated the effectiveness of a substance abuse prevention programme with a social norm correction. Results showed “a persistent positive effect over 18 months for alcohol abuse and for cannabis use, but not for cigarette smoking” [23]. The authors explained the lack of effect for smoking by stating that accumulating evidence illustrates how dependency in adolescence can occur following the sporadic use of tobacco. Cigarette smoking may also be a normative behaviour to a larger extent compared to episodes of drunkenness or illicit drug use in the age groups studied. However, this European study did not include Danish schools.

In Denmark, intervention studies based on social norms theory are sparse. A smaller scale study called the Ringsted trial, which partly used social norm approaches at primary schools, could demonstrate that the social norms intervention reduces misperceptions of descriptive norms related to smoking, alcohol, drug use, crime and bullying, but the sample size was too small to observe significant effects on behaviour [34]. Another intervention study using the social norms approach to reduce teenage smoking has been evaluated in the municipalities of Frederikssund and Bornholm among 6 graders, (“Alle de andre gør det”) resulting in some positive effects on perceptions. However, the study design did not allow for any conclusions on the effectiveness of the approach in Denmark [35]. Similarly, a qualitative survey in 2011 showed that 67 % of the Danish municipalities had adapted the social norms theory and tailored it to each of their school interventions. Unfortunately, neither of the designs allowed any measure of effectiveness [36].

The scientific evidence to date illustrates the need to strengthen effective interventions targeting school aged children in Denmark in order to positively influence their perceived and actual alcohol and other drug (AOD) use. As a result, this paper describes the design of the school-based cluster-randomized trial *The GOOD life* (In Danish: *Det GODE liv*) which aims to reduce AOD use and related harm among Danish adolescents. The trial is funded as the research project “De gode liv mellem de unge” by the Danish foundation TrygFonden.

## Methods/Design

### Aims and objectives

The study aims to develop and implement a social norms based intervention and to evaluate its effectiveness in reducing alcohol and other drug (AOD) use as well as social misperceptions among adolescents in grades 8–9 in the Region of Southern Denmark.

The specific objectives are to:

1. Assess the descriptive and injunctive norms regarding alcohol and other drug use among Danish adolescents in grades 8–9.
2. Asses the effect of *The GOOD life* intervention on
  - Descriptive and injunctive norms
  - Binge drinking and alcohol related harm
  - Other drug use (tobacco and marijuana)
3. Conduct a process evaluation in order to assess the feasibility of the intervention in the Danish school context and the fidelity and reach of the different intervention components of *The GOOD life*

### School setting

The study will be conducted at municipal lower secondary schools in the Region of Southern Denmark. Southern Denmark is one of five regions in Denmark with around 1.2 million inhabitants (21 % of the Danish population) [37]. In Denmark, all children are entitled to free tuition at Danish public schools, which consists of one year pre-school followed by nine years of primary and lower secondary school. The vast majority (about 80 %) of children attend the municipal school and children automatically attend the school in the area of the family’s residence [38].

### *The GOOD life* intervention

The intervention has been developed by the project team based on experience from the Social Norms Intervention to Prevent Polydrug Use Study SNIPE [22, 28], the “Guide to Marketing Social Norms for Health Promotion in Schools and Communities” [39] and on consultancy by Social Sense (Social Sense Ltd., Manchester, UK), a company producing and delivering social norms campaigns at schools in the United Kingdom. Intervention components are pre-tested in one school class and feedback from students is used to optimize the interventions’ design and construction (logo, posters and presentations).

*The GOOD life* provides normative feedback through social norms messages specific for each participating school. Social norms messages are phrased to challenge potential overestimations of peer behaviour and attitudes towards alcohol and other drug use, and thereby correct misperceptions by contrasting the perceived behaviour of peers from their school with assessed peer alcohol and other drug use from the baseline data collection. Examples of social norms messages are: “9 out of 10 pupils in 8th grade at [school name] have NEVER tried smoking” or “76 % of pupils at [school name] believe that it is not okay to drink alcohol if it affects school.” Such messages are used throughout the three main components of *The GOOD life*, which are:

1. **A one-hour normative feedback session:** Schools receive feedback sessions for single classes or grades of their school on the theory of social norms and the discrepancy between perceived and actual consumption of alcohol and other drugs led by a trained moderator. The session is based on interaction with students and student participation, e.g. through a web-based poll, which displays what the group collectively assumes to be the true estimates of alcohol and other drug use. The actual results from the baseline data collection at the same school are presented using an approach similar to Killos et al., [40]. Discrepancies between perceived and actual descriptive and injunctive norms are discussed with students.
2. **Posters:** Each school receives 4–5 posters with school specific social norms messages. Teachers are asked to display them for the remaining period of the intervention after the normative feedback session.
3. **Web-based application:** Through posters placed in their classrooms, students are encouraged to open a web-based application on their computers or smartphones, where they can test their social misunderstandings and receive information on the actual behavioural norms at their school. The poster displays the web-address as well as a QR code for direct access to the application. The web-based application functions similar to the web-poll in component 1, but on an individual basis and with social norms messages that differ in content from the messages presented in component 1 or 2. The web-based application has been developed by Social Sense Ltd. and was adapted for use at Danish schools in cooperation with the project team.

At each school, *The GOOD life* intervention starts with the normative feedback session conducted either in single classes or whole grades according to school preferences, followed by the posters, and lastly by the web-based application. The entire campaign lasts for approximately an 8 week period.

## Study design

**Fig. 1:**
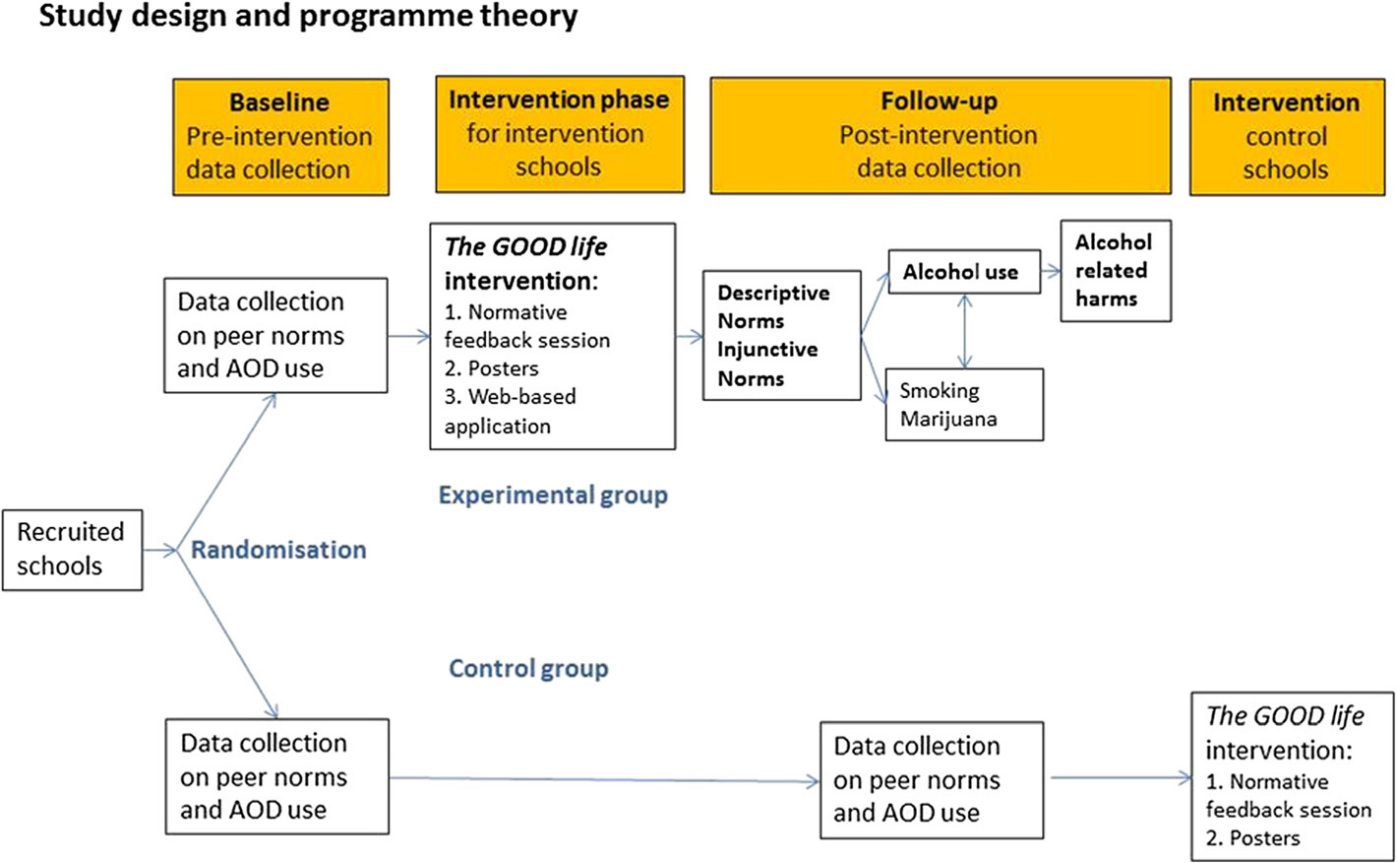
Study design and hypothesised causal relationships between the outcomes of the trial The GOOD life. Main outcomes to be studied are bolded

The study is designed as a cluster-randomized controlled trial with baseline data collection (T0) among students and follow-up data collection (T1) 3 months after baseline (Fig. 1). The trial is conducted from March 2015 to December 2016 among students in grades 8 or 9 at lower secondary public schools in the Region of Southern Denmark. Both intervention and control schools are recruited for the trial either for the spring term (with T0 in March and T1 in June) or for the autumn term (with T0 in September and T1 in December). After completing baseline and follow-up data collection, the intervention components 1 and 2 are also offered to control schools. Both intervention and control schools receive a written report with results of the baseline data collection specific to their school. The trial is registered at Current Controlled Trials with study ID ISRCTN27491960.

## Sample size calculation

Stata version 14.1 was used to calculate the sample size and the calculation was based on an estimated intra-class correlation of 0.02 [41]. The calculation showed that in order to detect a 20 % difference between intervention and control schools in the prevalence of binge drinking at follow-up with 80 % power, 39 schools with 35 students per school (*N* = 1.400 students), who are randomly assigned to intervention and control arm, need to be included in the trial.

## Recruitment

During autumn 2014, spring 2015 and spring 2016, schools in the Region of Southern Denmark are contacted through email and invited to participate in the trial. Schools that wish to participate are included in the study in the consecutive school term and participating schools are randomly allocated to either intervention or control group using the Microsoft Excel randomizations function.

## Content of the baseline questionnaire

The questionnaire is based on questions from existing questionnaires used to assess adolescent and youth health risk behaviours in social norms studies. Questionnaire items and scales originate from the Health Behaviour in School aged Children Study HBSC, the Social Norms Intervention to Prevent Polydrug Use Study SNIPE [28], the Teen Norms Survey [39], and the MULD study [42]. Additional questions have been added if deemed necessary to provide further context.

### Socio-demographic information

Information on age, grade and gender is collected in the baseline questionnaire. As a measure of the socio-economic status of the family, the HBSC family affluence scale (FAS) is used to collect information on family affluence based on car ownership, personal bedroom for the child, dishwasher ownership, number of bathrooms and number of computers [43]. In accordance with the HBSC questionnaire, students are also asked “How well off do you think your family is?” with answering options on a five point scale from “Very well off” to “Not at all well off” [44].

### Health and life satisfaction

Students are asked to respond to questions related to their self-rated health, their attitude regarding school and their school performance according to their teacher’s assessment based on the HBSC questionnaire [44]. Life satisfaction is measured according to the MULD questionnaire [42] and the Strengths and Difficulties Questionnaire (SDQ) is applied in its Danish version [45].

### Alcohol use

When presented with questions regarding alcoholic drinks, students receive an explanation of the amounts and different types of alcohol corresponding to one drink which is illustrated by a chart. Students are asked if they ever drank at least one drink of alcohol, and have ever been drinking more than 5 drinks during the same occasion. According to the MULD questionnaire, students are asked to identify the amount of occasions they have been drinking alcohol, whether or not they have been drunk, and whether or not they have been drinking more than 5 drinks in the past 30 days [42]. According to the SNIPE, students are also asked to state the number alcoholic drinks they normally consume on a typical day of drinking alcohol and to identify the highest number of alcoholic drinks they have consumed in a single session in the past 30 days [28].

### Smoking

According to HBSC, students are asked if they have ever smoked tobacco, and if yes, how often they smoke tobacco and whether or not they have smoked tobacco in the past 30 days [44].

### Marijuana and other illicit drug use

Students are asked if they have ever used cannabis or marijuana, and if yes, how many times they have used cannabis or marijuana in the past 30 days. According to MULD, students are asked if they have used any illegal drugs other than cannabis or marijuana, for example amphetamine, ecstasy, heroin or cocaine [42].

### Alcohol related harm

Based on the MULD and SNIPE questionnaires, students are asked whether they have ever experienced unusual or adverse events (e.g. memory loss, injury) as a result of alcohol use in the past 30 days and are provided with 15 different options and permitted multiple responses [28, 42].

### Perceptions of rates of peer alcohol and other drug use (descriptive norms)

Students are asked to rate the percentage of fellow students in their grade whom they perceived to be involved in the following behaviours: drinking alcohol, been drunk, drinking 5 or more drinks at the same occasion, smoking, using marijuana, using other illegal drugs. Similarly to the Teen Norms questionnaire [39], students are also asked to complete these ratings for the past 30 days. In addition, according to SNIPE, students are asked to rate the amount of times most pupils (at least 51 %) have used alcohol in the past 30 days and how many alcoholic drinks they think most (at least 51 %) of pupils in their grade normally consume in a day when they drink alcohol [28].

### Attitudes towards personal and peer alcohol and other drug use (injunctive norms)

According to the SNIPE questionnaire, students are asked to state which of the options presented best describe their own and their peers’ attitudes towards: tobacco, alcohol, larger amounts of alcohol and cannabis or marijuana [28].

## Content of the follow-up questionnaire

In the follow-up questionnaire, the SDQ questionnaire is omitted and in addition to the items from the baseline questionnaire, questions related to the process evaluation are included. This section covers the extent to which students received the three components of *The GOOD life* intervention, student satisfaction and engagement, mediating and obstructing factors, as well as open-ended questions where students’ feedback on the intervention components was provided.

## Effect evaluation

The effect of the intervention will be studied based on the intention-to-treat principle. The main outcomes of the intervention trial are binge drinking (more than 5 drinks on one occasion) in the last 30 days and perceptions of peer binge drinking among peers of their school and grade.

As secondary outcomes, marijuana use in the last 30 days and smoking in the last 30 days will also be studied. Another secondary outcome will include the difference in perceptions between intervention and control pupils with regard to smoking and marijuana use among peers in one’s school and grade. The effect of the intervention on the number of alcohol related harms will also be assessed.

We plan to use random effect models to study differences between intervention and control pupils in both main and secondary outcomes at follow-up while adjusting for baseline values and potential confounding factors (e.g. age, gender). A multilevel approach will be used in each model to account for clustering from pupils attending the same school.

The dose received (1, 2 or all three components of the intervention) will be included in another analysis in order to study if larger doses of the social norms intervention will lead to larger effect sizes.

## Process evaluation

The process evaluation is conducted in all interventions schools and will be used for both formative and summative purposes. The retrieved information will make it possible to explain and interpret the programme’s outcome.

The process evaluation uses a comprehensive and systematic approach with emphasis on assessing fidelity, dose delivered, dose received, reach, recruitment and context. Consequently, it studies all three components of the intervention, completeness of the intervention, pupil satisfaction and exposure to the intervention alongside participation rate. This is done through two main elements:

1. Qualitative interviews
  7–10 focus groups are conducted at schools and each group consists of 6–8 pupils. The moderator for the focus group discussion will initially introduce themes through an object of which an undirected discussion will be formed. In the following, the discussion will be more directed towards evaluating the program through open-ended questions. The interviews are tape recorded with permission from participants.
  Semi-structured interviews with teachers and social norm instructors which use open-ended questions and are tape recorded with permission from the participants.
2. A survey is conducted among pupils at schools. The survey covers the extent of implementation, completeness of intervention, pupil satisfaction and engagement, mediating and obstructing factors and open-ended questions where pupils can share and elaborate on their opinions. These process evaluation questions are administered as part of the follow-up questionnaire.

## Discussion

Denmark has a considerably higher alcohol-attributed disease burden when compared with Sweden or Norway, countries with which Denmark shares a similar type of welfare state with relatively low social inequality [46]. Therefore, Denmark is in special need of providing evidence-based prevention and intervention methods for health promotion practitioners at the local level that have the potential to prevent alcohol and other drug use. Since harmful drug-use in adolescence predicts harmful consumption patterns later in life, early intervention during adolescence is warranted.

The social norms approach has the potential to contribute to reducing the use of AOD. There is overall convincing evidence of its effectiveness in adolescents and young adults, but studies in Scandinavia, particularly in Denmark, are sparse. The most cited study in this context, the Ringsted trial, did not possess a sufficient sample size to apply rigorous analytical procedures and was conducted in only one city in Denmark, thus having a reduced geographical coverage [34]. The present trial, *The GOOD life*, aims to overcome these shortcomings in study design by using a randomized controlled design with longitudinal follow-up and a population-based sample with larger geographical coverage and a sufficient number of schools and subjects.

The combination of communication channels and mode are expected to be more effective than a single component social norms intervention (such as only through interactive technology) for a number of reasons: Firstly, in adding different components the intervention period is prolonged, which is regarded as important for gaining sustainable effects [17]. Secondly, the combination of information channels and intervention components increase the chance for pupils to participate in at least one or two components and therewith minimize attrition due to missing school attendance. And thirdly, we expect a combination of components in order to optimally meet the different preferences pupils may have in communicating and receiving messages (active, passive and interactive with new technologies). The trial is accompanied by a thorough process evaluation that will reveal how Danish pupils respond to, perceive and interact with social norms messages and whether any reactions, negative attitudes, or mistrust towards the approach needs to be taken into account. The collection of such information is vital in making appropriate recommendations for a larger scale use of the approach. Effectiveness alone is not enough to justify the use of the intervention and its country-wide implementation as there also needs to be certainty that adolescents have positive attitudes, perceptions and emotional reactions towards the intervention. Ensuring such responses is essential in minimizing potential negative side effects such as exclusion or marginalisation of certain individuals or groups of pupils, provoking reactions such as fear, or stimulating mistrust within or between groups of pupils or between pupils and teachers and other authorities. We aim to elucidate these issues through focus group interviews with pupils and through open questions in the follow-up questionnaire where pupils can freely express their attitudes regarding the intervention and its different components.

However there are also some limitations in this trial design. The authors are aware that the relatively short follow-up period of 3 months and the lack of a second follow-up does not allow for testing the long-term effects of the intervention. Due to the fact that many pupils leave the public schools after the 9^th^ year and continue their education at other schools a long-term follow-up is not feasible without substantial loss to follow-up. We are also aware that the study population is limited to one out of the five regions in Denmark in order to save on transportation costs to schools, but we do not regard this as a substantial limitation as the trial will be conducted in both rural and urban schools within the Region of Southern Denmark.

In conclusion, *The GOOD life* will provide fresh insight on descriptive and injunctive norms regarding AOD use among Danish adolescents. Furthermore, we regard this trial as an important study for providing unique and essential knowledge on the feasibility, implementation, context and effectiveness of a newly developed social norms intervention in the Danish school context.
