# Erratum to: The GOOD life: Study protocol for a social norms intervention to reduce alcohol and other drug use among Danish adolescents

**DOI:** 10.1186/s12889-017-4444-z

**Published:** 2017-05-24

**Authors:** Christiane Stock, Lotte Vallentin-Holbech, Birthe Marie Rasmussen

**Affiliations:** Unit for Health Promotion Research, Department of Public Health, University of Southern Denmark, Esbjerg, Denmark

## Erratum

Following publication of this study design article [1], it has come to our attention that some terminology was inconsistently used in the article. The GOOD life trial uses a definition of binge drinking of “5 or more drinks on one occasion” in accordance with the ESPAD study [2]. Both in the abstract and in the methods section, when describing measures of alcohol use or outcomes, any formulation of “more than 5 drinks” becomes “5 or more drinks”. Also, when respondents were asked to identify the amount of occasions they have been drinking 5 or more drinks in the past 30 days, the binge drinking definition was not according to the MULD study [3], but according to the ESPAD study [2].
